# Multi-Omics Immune Interaction Networks in Lung Cancer Tumorigenesis, Proliferation, and Survival

**DOI:** 10.3390/ijms232314978

**Published:** 2022-11-29

**Authors:** Qing Ye, Justin Hickey, Kathleen Summers, Brianne Falatovich, Marieta Gencheva, Timothy D. Eubank, Alexey V. Ivanov, Nancy Lan Guo

**Affiliations:** 1 West Virginia University Cancer Institute, Morgantown, WV 26506, USA; 2 Department of Biochemistry, School of Medicine, West Virginia University, Morgantown, WV 26506, USA; 3 Department of Microbiology, Immunology, and Cell Biology, School of Medicine, West Virginia University, Morgantown, WV 26506, USA; 4 Department of Occupational and Environmental Health Sciences, School of Public Health, West Virginia University, Morgantown, WV 26506, USA

**Keywords:** non-small cell lung cancer, prognosis, diagnosis, CRISPR-Cas9/RNAi, drug screening, targeted therapy

## Abstract

There are currently no effective biomarkers for prognosis and optimal treatment selection to improve non-small cell lung cancer (NSCLC) survival outcomes. This study further validated a seven-gene panel for diagnosis and prognosis of NSCLC using RNA sequencing and proteomic profiles of patient tumors. Within the seven-gene panel, *ZNF71* expression combined with dendritic cell activities defined NSCLC patient subgroups (*n* = 966) with distinct survival outcomes (*p* = 0.04, Kaplan–Meier analysis). *ZNF71* expression was significantly associated with the activities of natural killer cells (*p* = 0.014) and natural killer T cells (*p* = 0.003) in NSCLC patient tumors (*n* = 1016) using Chi-squared tests. Overexpression of ZNF71 resulted in decreased expression of multiple components of the intracellular intrinsic and innate immune systems, including dsRNA and dsDNA sensors. Multi-omics networks of *ZNF71* and the intracellular intrinsic and innate immune systems were computed as relevant to NSCLC tumorigenesis, proliferation, and survival using patient clinical information and in-vitro CRISPR-Cas9/RNAi screening data. From these networks, pan-sensitive and pan-resistant genes to 21 NCCN-recommended drugs for treating NSCLC were selected. Based on the gene associations with patient survival and in-vitro CRISPR-Cas9, RNAi, and drug screening data, MEK1/2 inhibitors PD-198306 and U-0126, VEGFR inhibitor ZM-306416, and IGF-1R inhibitor PQ-401 were discovered as potential targeted therapy that may also induce an immune response for treating NSCLC.

## 1. Introduction

Non-small cell lung cancer (NSCLC) has the second-highest cancer incidence rate and the highest cancer mortality rate for both men and women [1]. The major histological subtypes of NSCLC are lung adenocarcinoma (LUAD, 40% of NSCLC cases), squamous cell carcinoma (LUSC, 25–30%), and large cell carcinoma (LCC, 5–10%). Each subtype represents a distinct prognosis for patients and informs treatment options [2,3]. According to the current NCCN standard of care [4], stage 1A NSCLC patients do not receive adjuvant therapy after surgery. Osimertinib is recommended for stage 1B patients with EGFR exon 19 deletion or L858R. Adjuvant therapy is recommended for patients in stage 1B with high-risk features, i.e., tumor size > 4 cm, poor differentiation, vascular invasion, wedge resection, visceral pleural involvement, and unknown lymph node status, stage 2, and above. Patients in stages 3 and 4 receive additional radiotherapy [5]. Programmed Death 1 (PD-1) and its ligand PD-L1 compromise anti-tumor immunity while maintaining peripheral tolerance [6]. Anti-PD-L1 antibodies have revolutionized cancer immunotherapy, including NSCLC treatment [7]. NCCN guidelines [4] recently changed to establish adjuvant anti-PD-L1 immunotherapy (atezolizumab) after chemotherapy as the standard of care for stages 2/3A NSCLC patients with PD-L1 > 1%, following the FDA approval [8]. Yet, resectable NSCLC has a 5-year mortality rate of 40% in stage 1, 66% in stage 2, and 85% in stage 3A because of recurrence [9,10,11,12,13]. At present, there are no accurate prognostic tests that predict post-surgical recurrence/metastasis or inform the clinical benefits of adjuvant therapies, including chemotherapy and immunotherapy, in early-stage NSCLC patients—a significant unmet clinical need.

We recently discovered a seven-gene (*ABCC4*, *CCL19*, *CD27*, *DAG1*, *FUT7*, *SLC39A8*, and *ZNF71*) signature that accurately predicts the risk of recurrence/metastasis in retrospective analyses of 1500 early-stage NSCLC patients for all histological subtypes, including clinical trials [14,15]. Employing novel artificial intelligence (AI) methods and confirmed with qRT-PCR using frozen tumors (*n* = 331) [14], our assay also predicts the clinical benefits of receiving adjuvant chemotherapy in both training and validation sets, including a clinical trial JBR.10. Results from our seven-gene panel were corroborated in The Cancer Genome Atlas (TCGA) cohort for risk stratification of early-stage NSCLC patients (*n* = 923) [15]. Within this seven-gene panel, CD27 is a target for immune checkpoint inhibitors (ICIs) [16], and anti-CD27 mAb is being tested as an adjuvant immunotherapy in phase I/II clinical trials for multiple tumor types with promising results [17,18]. Within the seven-gene panel, ZNF71 protein expression quantified with AQUA produced robust patient stratification in two separate NSCLC cohorts (*n* = 191) in tissue microarrays [14]. We previously reported that the *ZNF71 KRAB* isoform was associated with epithelial-to-mesenchymal (EMT) transition and poor prognosis in NSCLC patients [19].

ZNF71 is a member of a large family of KRAB zinc finger transcription factors, KRAB-ZNFs, which due to the presence of the KRAB domain function as transcriptional repressors. One of the main roles ascribed to KRAB-ZNFs is the repression of retrotransposon class repetitive elements (TEs) [20] that comprise up to 36% of the human genome [21]. Retro TEs are remnants of ancient invaded viruses, which could produce dsRNA molecules and RNA/DNA hybrids. Although the vast majority of TEs in the human genome are inactivated by mutations, a small number of full-length functional elements including long interspersed nuclear elements (LINEs) and human endogenous retroviruses (HERVs) are capable of retrotransposition, mimicking viral infection. While normally silenced, they could be reactivated in cancer or response to therapy [22,23]. The majority of cellular dsRNA is the result of the TEs transcription [24]. Non-degraded dsRNAs are recognized by specific proteins of the intracellular innate immune system also called pattern recognition receptors (PRRs), such as MDA5/IFIH1 and others, ultimately leading to a Type I interferon production [25]. Acting as specific dsRNA sensors downstream or in parallel with PRRs, the OAS-RNase L and the PKR/EIF2AK2 pathways degrade endogenous and viral dsRNA and block global cellular translation, respectively [26,27]. In addition, a growing number of host restriction factors of the intrinsic immune system can be engaged in an anti-viral response, including SAMHD1, TRIM5a, MX, and IFITM proteins [28].

Most human tumors display chromosomal instability (CIN) phenotype and aneuploidy, which are often accompanied by the generation of micronuclei and the presence of cytosolic dsDNA. Cytosolic dsDNA activates the cGAS-STING signaling pathway [29]. STING facilitates the activation of TBK1 kinase leading to its autophosphorylation on S172 [30] and subsequent phosphorylation of the IRF3 transcription factor, which in turn activates interferon response genes.

In this study, we sought to (1) further evaluate the diagnostic and prognostic implications of the seven-gene panel using both RNA-sequencing and proteomic profiles in diverse NSCLC patient cohorts and examine the associated immune cell activities during NSCLC tumorigenesis and progression; (2) investigate the functional involvement of *ZNF71 KRAB* in innate immunity; (3) identify molecular networks mediated by *ZNF71* relevant to innate immunity in NSCLC tumors and normal adjacent lung tissues using a novel AI technology based on Boolean implication networks; (4) discover pan-sensitive and pan-resistant genes to a panel of 21 NCCN recommended drugs for treating NSCLC from the above identified molecular association networks; and (5) explore therapeutic compounds as new or repositioning drugs for treating NSCLC for designed mechanisms of actions based on our analysis of patient tumor profiles and in-vitro CRISPR-Cas9/RNAi and drug screening data using Connectivity Map (CMap) [31,32].

## 2. Results

### 2.1. Further Validation of the Seven-Gene Signature in Prognosis for NSCLC

Our previous work [14] developed a prognostic and predictive seven-gene assay including *ABCC4*, *CCL19*, *CD27*, *DAG1*, *FUT7*, *SLC39A8*, and *ZNF71* for early-stage NSCLC. In this study, we further explored the prognostic capacity of the seven-gene signature using proteomic profiles in a Chinese LUAD cohort from Xu et al. [33] (*n* = 103) and TCGA-NSCLC datasets (*n* = 923, TCGA-LUAD and TCGA-LUSC combined) of patient samples with sufficient survival information. Immune cell-type activities associated with different prognostic patient groups were investigated.

**Figure 1:**
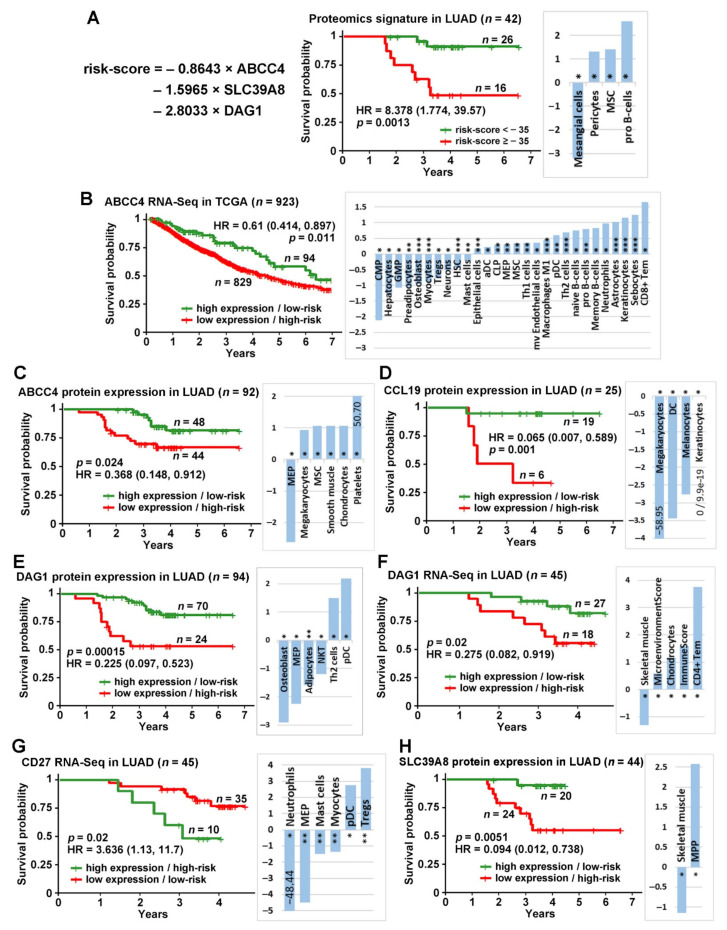
The prognosis of the seven-gene panel using RNA sequencing and proteomic profiles in NSCLC tumors. Kaplan–Meier analysis and log_2_ ratio of xCell scores of the high-risk group vs. the low-risk group using: (**A**) three-gene (ABCC4, SLC39A8, and DAG1) Cox model in LUAD proteomics data; (**B**) RNA sequencing data of *ABCC4* in TCGA-NSCLC; (**C**) proteomics data of ABCC4 in LUAD [33]; (**D**) proteomics data of CCL19 in LUAD [33]; (**E**) proteomics data of DAG1 in LUAD [33]; (**F**) RNA sequencing data of *DAG1* in LUAD [33]; (**G**) RNA sequencing data of *CD27* in LUAD [33]; (**H**) proteomics data of SLC39A8 in LUAD [33]. Two sample *t*-tests were performed to test the difference between the two groups (*: *p* < 0.05; **: *p* < 0.01; ***: *p* < 0.001).

ABCC4, CCL19, CD27, DAG1, and SLC39A8 from the seven-gene assay were available in log_10_ transformed proteomics data of Xu’s LUAD cohort [33]. A multivariate Cox model was built based on these five genes to calculate the coefficients for the risk score. A stepwise model selection that dropped the least significant variable in each iteration was used to reach an optimal model. The final risk-score equation was shown in Figure 1A. A risk-score cutoff point of −35 was found to stratify the patient samples with significantly different survival outcomes. The Kaplan–Meier analysis results showed that the patients with a risk score lower than −35 had significantly better survival outcomes than the patients with a risk score higher than −35 in Xu’s LUAD proteomics data [33] (*p* = 0.0013, HR: 8.378 [1.774, 39.57]; Figure 1A).

The Kaplan–Meier analysis results also showed significant stratifications for each of these five genes (ABCC4, CCL19, CD27, DAG1, and SLC39A8) in RNA-sequencing/proteomic profiling in Xu’s LUAD [33] or TCGA-NSCLC patient cohorts. Patients with a higher expression of *ABCC4* (cutoff = 10.45) in TCGA-NSCLC RNA-sequencing data survived significantly longer than those with a lower expression of *ABCC4* (Figure 1B). Patients with a higher expression of ABCC4 (cutoff = 6.22; Figure 1C), CCL19 (cutoff = 6.31; Figure 1D), DAG1 (cutoff = 6.72; Figure 1E), and SLC39A8 (cutoff = 6.48; Figure 1H) in log_10_ transformed proteomics data in Xu’s LUAD cohort survived significantly longer than those with a lower protein expression of these genes. Patients with a higher expression of *DAG1* (cutoff = 3.9; Figure 1F) in Xu’s LUAD RNA sequencing data [33] survived significantly longer than patients with a lower expression of *DAG1*. Patients with a higher expression of *CD27* (cutoff = 8.72; Figure 1G) in Xu’s LUAD RNA sequencing data [33] survived a significantly shorter time than patients with a lower expression of *CD27.* ZNF71 and FUT7 did not have any protein expression measurements in Xu’s LUAD cohort [33]. In mRNA expression profiles of the TCGA NSCLC and Xu’s LUAD [33] cohorts, *ZNF71* and *FUT7* were not significantly associated with patient survival outcomes.

The xCell scores were computed for each patient sample in TCGA-NSCLC and Xu’s LUAD [33] cohorts with the corresponding RNA sequencing data. For each significant patient stratification in survival analysis, immune cell types with a significant difference in activities (two-sample *t*-tests; *p* < 0.05) between high-risk and low-risk patient tumors were identified. The log_2_ ratio of xCell scores between high-risk vs. low-risk tumors was shown in Figure 1 for each stratification. A positive log_2_ (xCell score) indicates that cell type activity varies more in high-risk tumors; a negative log_2_ (xCell score) indicates that cell type activity varies more in low-risk tumors.

**Figure 2:**
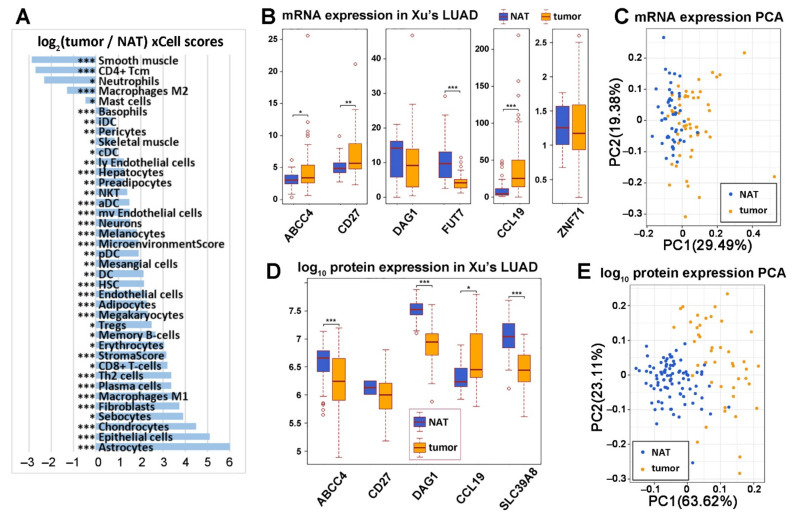
Classification of non-cancerous adjacent tissues (NATs) and lung adenocarcinoma (LUAD) tumors with the seven-gene panel. (**A**) Log_2_ ratio of xCell scores of the LUAD tumor vs. NATs RNA sequencing data. Two sample *t*-tests were performed to test the difference between the two groups (*: *p* < 0.05; **: *p* < 0.01; ***: *p* < 0.001). Boxplots of available RNA sequencing gene expression (**B**) and protein expression (**D**) in tumors and NATs of the 7-gene panel in LUAD patients [33]. Principal component analysis (PCA) of RNA sequencing gene expression (**C**) and protein expression (**E**) in separating NATs and LUAD tumors in patients.

The log_2_ ratios of significantly different xCell scores between Xu’s LUAD tumors and their paired non-cancerous adjacent tissues (NATs) [33] were also shown in Figure 2A. The following cell types had more varied levels in NATs than in tumors: smooth muscle, CD4+ central memory T cells (Tcm), neutrophils, macrophages M2, and mast cells. The following cell types had more varied levels in tumors than in NATs: basophils, lentivirus-induced dendritic cells (IDC), pericytes, skeletal muscle, conventional dendritic cells (cDC), ly endothelial cells, hepatocytes, natural killer T cells (NKT), activated dendritic cells (aDC), mv endothelial cells, neurons, melanocytes, microenvironment score (the sum of all immune and stromal cell types), plasmacytoid dendritic cells (pDC), mesangial cells, dendritic cells, hematopoietic stem cells (HSC), endothelial cells, adipocytes, megakaryocytes, Tregs, memory B-cells, erythrocytes, StromaScore (the sum of adipocytes, fibroblasts, and endothelial cells), CD8+ T-cells, Th2 cells, plasma cells, macrophages M1, fibroblasts, sebocytes, chondrocytes, epithelial cells, and astrocytes. 

### 2.2. Diagnostic Implication of the Seven-Gene Signature in NSCLC

We examined the potential of using the seven-gene panel to separate NSCLC tumors from NATs. Within the seven-gene panel, there were six genes (*ABCC4*, *CD27*, *DAG1*, *FUT7*, *CCL19*, and *ZNF71*) available in the RNA sequencing data of Xu’s LUAD cohort [33] (Figure 2B). The principal component analysis (PCA) using the mRNA expression of these six genes to separate tumors and NATs in Xu’s LUAD cohort [33] is shown in Figure 2C. There were five genes (ABCC4, CCL19, CD27, DAG1, and SLC39A8) available in Xu’s LUAD proteomics data [33] (Figure 2D). The separation of LUAD tumors and NATs using these five protein expression data is shown in Figure 2E. *ABCC4* was expressed significantly higher in LUAD tumors than NATs in RNA sequencing data (*p* < 0.05, two sample *t*-tests, Figure 2B) but was expressed significantly higher in NATs than in tumors in proteomics data (*p* < 0.001, two sample *t*-tests, Figure 2D). *CCL19* was expressed significantly higher in LUAD tumors than in NATs in both RNA sequencing and proteomics data (*p* < 0.05, two sample *t*-tests, Figure 2B,D). *CD27* was expressed significantly higher in LUAD tumors in RNA sequencing data (*p* < 0.01, two sample *t*-tests, Figure 2B) but was not significantly different in protein expression between LUAD tumors and NATs (Figure 2D). DAG1 and SLC39A8 were expressed significantly higher in NATs in LUAD proteomics data (*p* < 0.001, two sample *t*-tests, Figure 2D).

To evaluate the accuracy of using the seven-gene panel in classifying tumors from NATs in Xu’s LUAD cohort [33], we applied seven commonly used machine-learning classification algorithms. These algorithms included decision tree, k-nearest neighbor (KNN), logistic regression, Naïve Bayes, random forests, support vector machine (SVM), and radial basis function (RBF) network. Classification methods were performed in Weka with 10-fold cross-validation. In LUAD RNA sequencing data [33], six genes (*ABCC4*, *CCL19*, *CD27*, *DAG1*, *FUT7*, and *ZNF71)* with available mRNA expression data were used in the classification. Random forests and RBF networks had the highest overall classification accuracy of 0.86. The random forests classification had a sensitivity of 0.882, a specificity of 0.837, a ROC of 0.927, and an odds ratio of 38.44 in the 10-fold cross-validation of tumors vs. NATs (*n* = 51). The RBF network had a sensitivity of 0.824, a specificity of 0.898, a ROC of 0.896, and an odds ratio of 41.07 in the 10-fold cross-validation of tumors vs. NATs. Three genes (ABCC4, DAG1, and SLC39A8) with available protein expression data were used in the classification. SVM had the highest overall classification accuracy of 0.941, with a sensitivity of 0.867, a specificity of 0.978, a ROC of 0.922, and an odds ratio of 286 in classifying LUAD tumors from NATs (*n* = 103). Overall, the 7-gene panel generated high accuracy in separating tumors from NATs using RNA-sequencing or proteomic profiles, indicating its diagnostic implications in NSCLC. Detailed information on the classification results is included in Tables S1 and S2 in Supplementary File S1.

### 2.3. ZNF71 Expression and Selected Immune Cells in NSCLC Prognosis

ZNF71 protein expression was not available in Xu’s LUAD cohort [33]. In our previous study, ZNF71 protein expression quantified with AQUA was associated with a good prognosis in NSCLC patients (*n* = 191) in tissue microarrays [14]. Although *ZNF71* overall mRNA expression was not associated with NSCLC patient survival outcomes, *ZNF71 KRAB*, the transcriptional repression isoform, was an independent poor prognostic factor in early-stage NSCLC [19]. Furthermore, *ZNF71 KRAB* was associated with EMT in NSCLC patient tumors (*n* = 197) and epithelial cell lines (*n* = 117). ZNF71 protein expression positively correlated with epithelial markers E-cadherin and Cytokeratin and negatively correlated with mesenchymal markers ZEB1 and Vimentin in Western blots [15], consistent with the association between ZNF71 protein expression and favorable patient prognosis [19]. Based on the function of structurally relevant KRAB-ZNFs, we hypothesized that *ZNF71* could be involved in the suppression of endogenous transposable elements (TEs) expression, which is often activated in cancer and can trigger an innate immune response.

**Figure 3:**
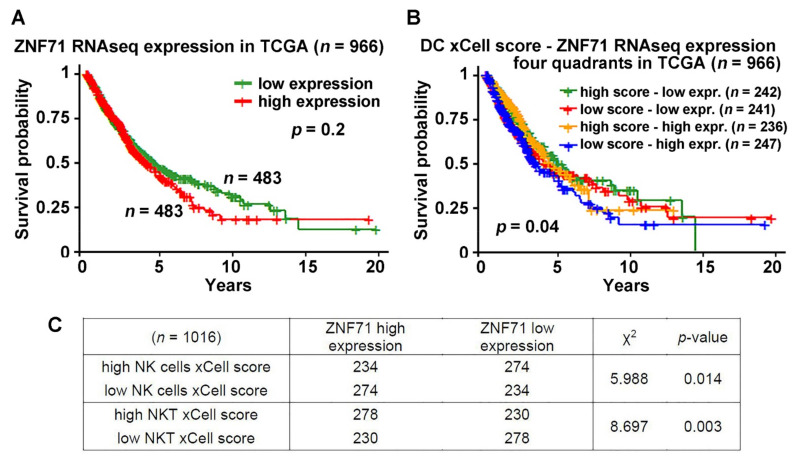
Association of *ZNF71* and xCell scores of selected immune cells. Prognostic implications of *ZNF71* and xCell scores of dendritic cells in TCGA NSCLC patient tumors. (**A**) Kaplan–Meier analysis of TCGA-NSCLC patients stratified by the median value of *ZNF71* mRNA expression. (**B**) Kaplan–Meier analysis of TCGA-NSCLC patients stratified by the median value of *ZNF71* mRNA expression and the median value of dendritic cells (DC) xCell scores. (**C**) χ^2^ test results of *ZNF71* expression vs. xCell scores of NK cells and NKT in TCGA-NSCLC patient tumors.

Next, we tested if *ZNF71* expression and specific immune cell activities have any associations with NSCLC prognosis. The Kaplan–Meier analysis results showed that if we used the median value of *ZNF71* mRNA expression as the cutoff to stratify TCGA-NSCLC, the low-expression group and high-expression group did not have a significant difference in the survival outcomes (Figure 3A). When we included the dendritic cell (DC) xCell score and created a four quadrants stratification with the median of both *ZNF71* mRNA expression and DC xCell score, i.e., high DC xCell score–low *ZNF71* expression, low DC xCell score–low *ZNF71* expression, high DC xCell score–high *ZNF71* expression, and low DC xCell score–high *ZNF71* expression, the Kaplan–Meier analysis results showed a significant difference in survival among the four groups (log-rank *p* = 0.04, Figure 3B). These results showed that patients with different *ZNF71* expressions and DC activities had distinct survival outcomes. Those with high DC xCell scores (representing more varied DC levels) and low *ZNF71* expression had the best survival outcomes, whereas those with low DC xCell scores (representing less varied DC levels) and high *ZNF71* expression had the worse survival outcomes (Figure 3B). Out of all available cell types analyzed with xCell, the DC xCell score is the only metric that can generate significant prognostic stratification when combined with *ZNF71* gene expression in TCGA NSCLC patients. Furthermore, the *ZNF71* expression level had significant associations with the xCell scores of natural killer (NK) cells and NKT in TCGA NSCLC patient tumors (*p* < 0.01, χ^2^ tests, Figure 3C). These results are consistent with a proposed model that tumor-derived substances trigger the early generation of IFN-β by host CD11c+ DCs. The cross-presentation of tumor-derived antigens is then stimulated by this IFN-β acting on the CD8α+ DC subset, resulting in the cross-priming of CD8+ T cells specific for the tumor antigen. These reactivated T lymphocytes may then move toward the tumor and cause more tumor cell death [34].

### 2.4. Overexpression of ZNF71 KRAB, KRAB-Less Isoforms Suppresses Innate Immune Response

**Figure 4:**
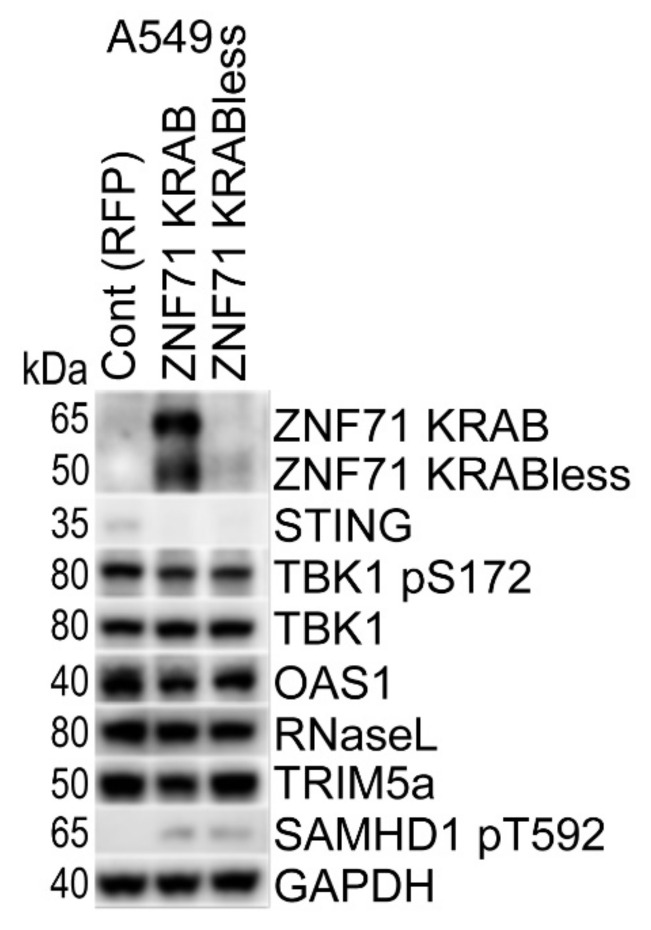
ZNF71 suppresses components of the innate and intrinsic immune response. Western blotting of control RFP, ZNF71 KRAB, and KRABless isoforms overexpression, as well as of several markers of intracellular innate and intrinsic immune systems in A549 cells. GAPDH is shown as the loading control.

To investigate the potential function of ZNF71 in intracellular immune response, we overexpressed its KRAB and KRAB-less isoforms in lung adenocarcinoma A549 cells (Figure 4). The latter is missing the KRAB domain (as a result of spliced-out exon 3) and encodes an approximately 55kDa protein. Interestingly, ZNF71 overexpression led to the downregulation of STING and its downstream effector S172-phosphorylated TBK1, while the total TBK1 level was not decreased. Similarly, we observed downregulation of OAS1, while RNase L expression was not significantly changed. Two viral restriction factors were either downregulated, TRIM5a, or inactivated by phosphorylation, SAMHD1 pT592 (Figure 4). These data suggest that overexpression of ZNF71 results in decreased expression of multiple components of the intracellular intrinsic and innate immune systems, including dsRNA and dsDNA sensors. Although direct targets of ZNF71 have not been identified yet, these results are consistent with a hypothesis that ZNF71 suppresses the transcription of genomic TEs.

### 2.5. Gene Association Networks of ZNF71 and Intracellular Innate Response Genes

**Figure 5:**
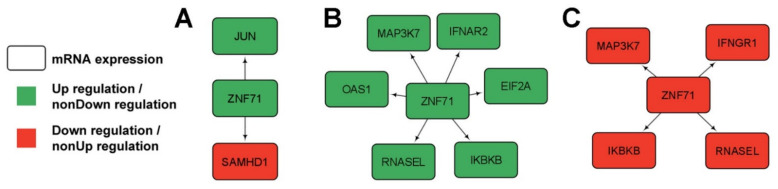
Direct gene co-expression networks of *ZNF71* and intracellular innate immune response (IIIR) genes in lung adenocarcinoma (LUAD) patient samples using RNA sequencing data. (**A**) Direct gene associations of *ZNF71* and IIIR genes when *ZNF71* is upregulated in LUAD tumors. (**B**) Direct gene associations of *ZNF71* and IIIR genes when *ZNF71* is upregulated in NATs. (**C**) Direct gene associations of *ZNF71* and IIIR genes when *ZNF71* is downregulated in NATs.

To explore gene interactions and pathways among *ZNF71* and the intracellular intrinsic and innate immune systems, multi-omics association networks involving *ZNF71* and genes examined in Western blot (Figure 4) were computed with the Boolean implication network method in Xu’s LUAD cohort [33] containing both mRNA and protein expression profiles in tumors and NATs. The intracellular innate immune response (IIIR) genes included (1) interferons and their receptors: *IFNA16*, *IFNA17*, *IFNA21*, *IFNA22P*, *IFNA4*, *IFNA5*, *IFNAR1*, *IFNAR2*, *IFNE*, *IFNG*, *IFNG-AS1*, *IFNGR1*, *IFNGR2*, *IFNK*, *IFNL1*, *IFNL3*, *IFNLR1*, *IFNW1*; (2) the cGAS-STING pathway: *CGAS*, *TMEM173/STING1*, *TBK1*, *IKBKB*, *IRF3*, *IRF7*, *AIM2*, maybe *JUN*, and *MAP3K7*; (3) the OAS-RNase L pathway: *OAS1* and *RNASEL;* (4) viral restriction factors: *SAMHD1* and *TRIM5*; (5) cyclin-dependent kinase: *CDK1*; (6) the co-repressor for KRAB-ZNFs: *TRIM28*; and (7) the housekeeping glycolysis gene: *PFKL*. For the seven-gene panel and the IIIR genes, the status of proliferation as measured in CRISPR-Cas9/RNAi in NSCLC cell lines and differential expression analysis in each studied patient cohort was included in Supplementary File S1 Table S3. First, direct mRNA co-expression networks of *ZNF71* and IIIR genes (*p* < 0.05, *z* tests) were found when (1) *ZNF71* was up-regulated in LUAD tumors (Figure 5A), (2) *ZNF71* was up-regulated in NATs (Figure 5B), and (3) *ZNF71* was down-regulated in NATs (Figure 5C) in the analysis of RNA-sequencing data from Xu et al. [33]. No significant direct gene co-expression relations were found between *ZNF71* and IIIR genes when *ZNF71* was downregulated in LUAD tumors.

The computed direct gene associations between *ZNF71* and IIIR genes (Figure 5) did not provide sufficient information to infer signaling pathways relevant to *ZNF71*-mediated innate immune responses. Here, the computationally derived gene associations do not represent the biological interactions one would expect to observe in genome-scale profiling after *ZNF71* overexpression/knockdown. To infer pathways and interactions between *ZNF71* and IIIR genes, we expanded the gene association networks as follows.

**Figure 6:**
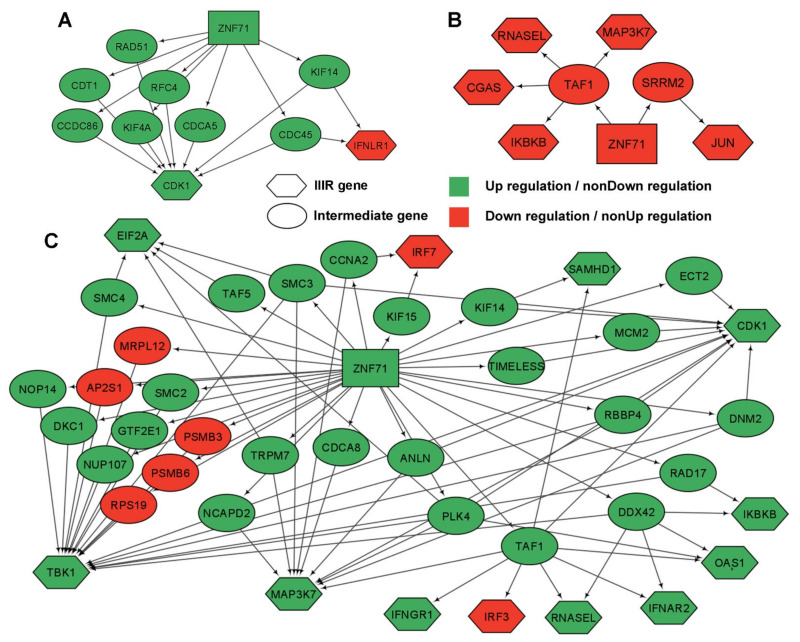
Indirect gene co-expression networks of *ZNF71* and intracellular innate immune response (IIIR) genes in lung adenocarcinoma (LUAD) patient samples using RNA sequencing data. (**A**) Gene associations of *ZNF71* with IIIR genes through intermediate genes in LUAD tumors when ZNF71 is upregulated. (**B**) Gene associations of ZNF71 with IIIR genes through intermediate genes in NATs when ZNF71 is downregulated. (**C**) Gene associations of *ZNF71* with IIIR genes through intermediate genes in NATs when *ZNF71* is upregulated. *ZNF71* is in a rectangle.

Second, we also identified indirect gene association networks between *ZNF71* and IIIR genes using RNA sequencing data from Xu et al. [33]. *ZNF71* had co-expression associations with IIIR genes (*p* < 0.05, *z* tests) through some intermediate genes. To form a manageable size of networks, we filtered the intermediate genes with the following criteria: (1) the gene was differentially expressed between LUAD tumors vs. NATs (*p* < 0.05, two-sample *t*-tests); (2) the gene was a proliferation gene that had a significant effect (dependency score < −0.5) on 50% or more NSCLC cell lines in RNAi or CRISPR-Cas9 screening data; (3) the gene was a prognostic gene that can significantly stratify the patient survival in RNA sequencing data of both TCGA-LUAD and Xu’s LUAD [33] patient cohorts. The indirect gene association networks with intermediate genes that meet all three criteria were found when *ZNF71* was up-regulated in LUAD tumors (Figure 6A), when *ZNF71* was down-regulated in NATs (Figure 6B), and when *ZNF71* was up-regulated in NATs (Figure 6C). Again, no significant indirect gene co-expression relations were found between *ZNF71* and IIIR genes when *ZNF71* was downregulated in LUAD tumors.

**Figure 7:**
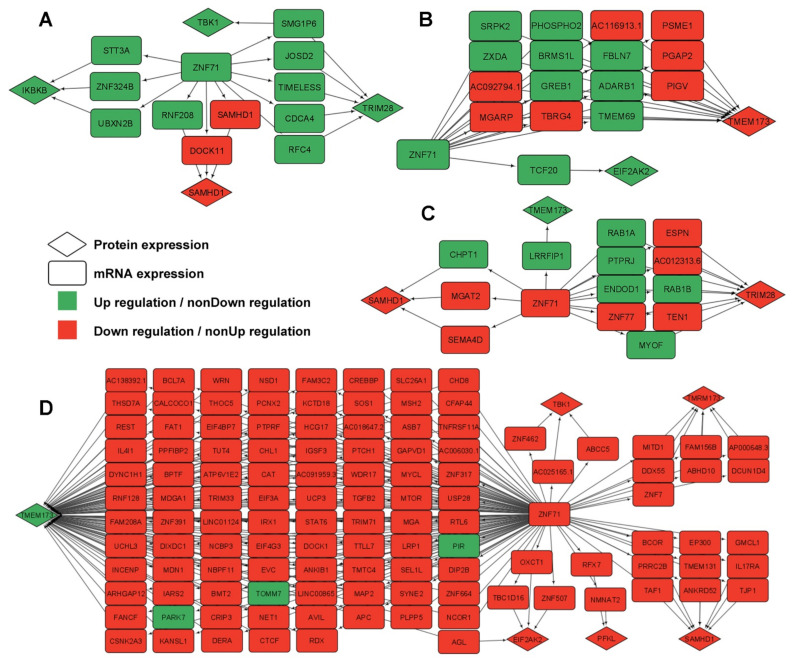
Multi-omics gene association networks of *ZNF71* and intracellular innate immune response (IIIR) genes in lung adenocarcinoma (LUAD) patient samples using RNA sequencing and proteomics data. (**A**) Gene associations between *ZNF71* and IIIR genes through intermediate genes in LUAD tumors when *ZNF71* is upregulated. (**B**) Gene associations between *ZNF71* and IIIR genes through intermediate genes in NATs when *ZNF71* is upregulated. (**C**) Gene associations between *ZNF71* and IIIR genes through intermediate genes in LUAD tumors when *ZNF71* is downregulated. (**D**) Gene associations between *ZNF71* and IIIR genes through intermediate genes in NATs when *ZNF71* is downregulated.

Third, although ZNF71 was not available in LUAD proteomics data from Xu et al. [33], *ZNF71* mRNA expression had indirect associations with the protein expression of some IIIR genes through intermediate genes. The indirect gene association networks of *ZNF71* (mRNA expression) → intermediate genes (mRNA expression) → IIIR genes (protein expression) were found in LUAD tumors when *ZNF71* was up-regulated (Figure 7A) and when *ZNF71* was down-regulated (Figure 7C), in NATs when *ZNF71* was up-regulated (Figure 7B), and when *ZNF71* was down-regulated (Figure 7D).

We examined the pathways in *ZNF71* co-expression networks with ToppGene. Genome-scale *ZNF71* co-expression networks containing all the genes with a significant association (*p* < 0.05, *z* tests) with *ZNF71* were constructed with the Boolean implication networks using RNA sequencing data in LUAD tumors and NATs from Xu et al. [33], respectively. For each disease state, the significantly (*p* < 0.05) enriched pathways of the gene co-expression networks when *ZNF71* was upregulated or downregulated were compared. There were 30 common pathways between the networks when *ZNF71* was upregulated or downregulated in NATs, focusing on DNA repair, RTK signaling, and transcriptional regulation (Supplementary File S2). There were no common pathways between the networks when *ZNF71* was upregulated or downregulated in LUAD tumors. Of the 30 common pathways in NATs, a generic transcription pathway (ToppGene ID: 1269650) and gene expression (ToppGene ID: 1269649) were present in the pathways when *ZNF71* was upregulated in LAUD tumors; whereas membrane trafficking (ToppGene ID: 1269877) and vesicle-mediated transport (ToppGene ID: 1269876) showed up in the pathways associated with a *ZNF71* downregulated network in LUAD tumors. Once we focused the gene co-expression networks on *ZNF71* and IIIR genes when *ZNF71* was upregulated or downregulated, 21 common pathways relevant to immune response were found in LUAD tumors; two common pathways, gene expression and generic transcription pathways, were found in NATs. Detailed significantly (*p* < 0.05) enriched pathways associated with *ZNF71* genome-wide co-expression networks, indirect networks of *ZNF71* and IIIR genes without any filtering criteria applied, and networks shown in Figure 6 and Figure 7 are included in Supplementary File S2.

### 2.6. Identification of Genes Associated with Drug Response

A total of 21 NCCN-recommended drugs for systemic or targeted therapy for treating NSCLC were available in the Cancer Cell Line Encyclopedia (CCLE) drug screening data. We sought to identify pan-sensitive and pan-resistant genes to these 21 drugs using CCLE RNA sequencing and proteomics profiles in human NSCLC cell lines. The following genes were included in the drug-sensitivity analysis: (1) the seven-gene panel, (2) selected epithelial genes (*CDH1*, *EPCAM*, *ESRP1*, *ESRP2*, *DDR1*, *CTNNB1*, *CD24*, *CLDN7*, *KRT8*, *KRT19*, and *RAB25*) and mesenchymal genes (*ZEB1*, *VIM*, and *FN1*), (3) IIIR genes (Figure 4), and (4) all the genes in the *ZNF71*-IIIR gene association networks (Figure 5, Figure 6 and Figure 7). Genes that were expressed significantly higher (*p* < 0.05; two-sample *t*-tests) in sensitive NSCLC cell lines for a specific drug were defined as sensitive genes. The epithelial and mesenchymal genes were included because the *ZNF71 KRAB* isoform was associated with EMT, and a 14-gene EMT classifier containing these genes separated early-stage NSCLC patients into distinct prognostic groups with disparate survival outcomes [19]. For a specific drug, genes that were expressed significantly higher (*p* < 0.05; two-sample t-tests) in resistant NSCLC cell lines were defined as resistant genes. In this study, we only selected the genes that were pan-sensitive or pan-resistant (Table 1 and Table 2). Pan-sensitive genes were the genes that were identified as either sensitive or not resistant to all the studied 21 drugs. Similarly, pan-resistant genes were the genes that were identified as either resistant or not sensitive to all the studied 21 drugs. PRISM [35] and GDSC1/2 [36,37,38] drug screening data were included in this analysis.

### 2.7. Functional Pathways Associated with the ZNF71 Co-Expression Networks and Discovery of Therapeutic Targets

To discover functional pathways and therapeutic targets to improve NSCLC treatment outcomes, pan-sensitive (*n* = 23) and pan-resistant (*n* = 31) genes in RNA sequencing data (Table 1) were used as CMap input candidate genes. The up-regulation of pan-sensitive genes and down-regulation of pan-resistant genes are expected to enhance NSCLC treatment response. Thus, the pan-sensitive genes and the pan-resistant genes were used as the initial up- and down-regulated gene lists in the CMap, respectively. The following steps were further applied to the gene lists to inhibit NSCLC proliferation, reverse EMT, and induce immune response: (1) excluding proliferation genes that had a significant effect (dependency score < −0.5) on at least 50% of NSCLC cell lines in both CRISPR-Cas9 and RNAi from the up-regulated gene list; (2) excluding survival protective genes (*p* < 0.05, HR < 1: univariate Cox model in RNA sequencing data of Xu’s LUAD cohort [33] and TCGA-NSCLC data) from the down-regulated gene list; (3) excluding the hazard genes (*p* < 0.05, HR > 1; univariate Cox model in RNA sequencing data of Xu’s LUAD cohort [33] and TCGA-NSCLC data) from the up-regulated gene list; (4) excluding the mesenchymal genes from the up-regulated gene list; (5) excluding epithelial genes from the down-regulated gene list; and (6) adding *CD27*, *PD1 (PDCD1)*, and *PDL1 (CD274)* in the downregulated gene list.

**Figure 8:**
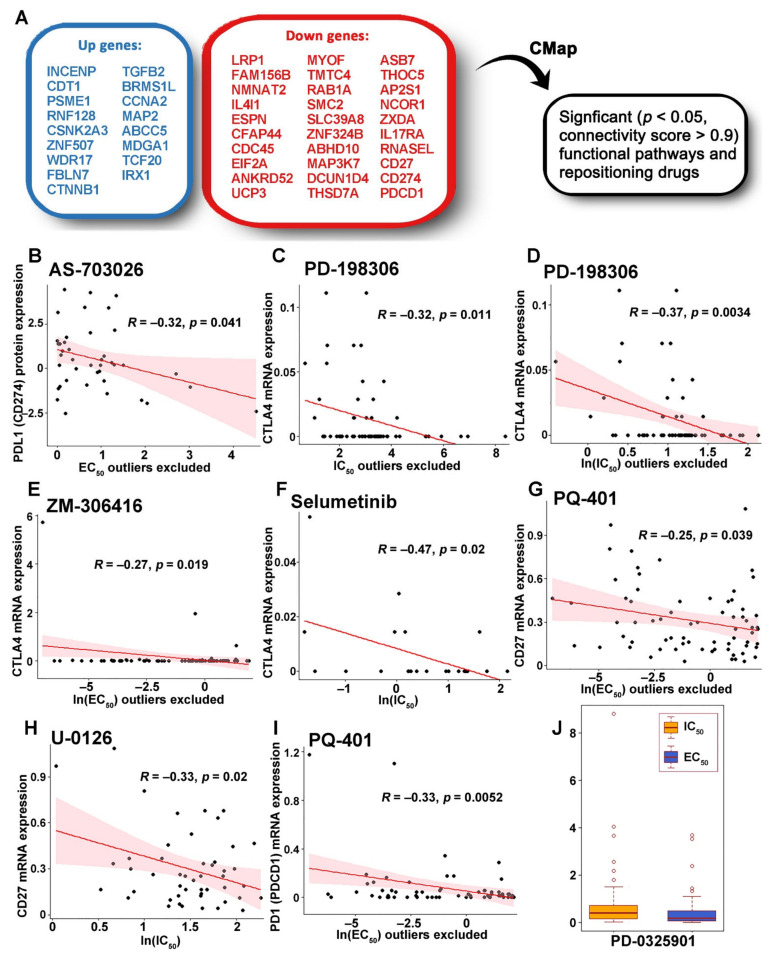
Discovering repositioning drugs based on the selected genes: Selection of significant functional pathways and repositioning drugs based on the selected genes (**A**). The Pearson correlations of PDL1 (CD274) protein expression with AS-703026 EC_50_ excluding outliers (**B**), *CTLA4* mRNA expression with PD-198306 IC_50_ excluding outliers (**C**), PD-198306 ln(IC_50_) excluding outliers (**D**), ZM-306416 ln(EC_50_) excluding outliers (**E**), and selumetinib ln(IC_50_) (**F**), *CD27* mRNA expression with PQ-401 ln(EC_50_) excluding outliers (**G**) and U-0126 ln(IC_50_) (**H**), *PD1*(*PDCD1*) mRNA expression with PQ-401 ln(EC_50_) excluding outliers (**I**). Selected compounds that had a low average IC_50_ and EC_50_ in the CCLE NSCLC cell lines (*n*= 64 [IC_50_]; *n* = 88 [EC_50_]) (**J**).

With the final up- and downregulated genes (Figure 8A) as CMap input, significantly enriched (*p* < 0.05, connectivity score > 0.9) functional pathways (Supplementary File S1 Table S4), compound sets (Supplementary File S1 Table S5), and 28 potential new or repositioning drugs were identified. In addition to PDL1 and CD27, other immune checkpoint inhibitors (ICIs) for treating advanced metastatic NSCLC include anti-PD-1 nivolumab and anti-CTLA4 ipilimumab [39]. To confirm the compound inhibitory effects on ICIs, we further checked if the compounds had a significant negative correlation (*p* < 0.05, *R* < 0, Pearson’s correlation test) between drug concentration and mRNA or protein expression of major ICIs for NSCLC treatment, including CD27, CTLA4, PD1 (PDCD1), and PDL1 (CD274) in the CCLE NSCLC cell lines (*n* = 135). EC_50_ of AS-703026 had a significant negative correlation with PDL1protein expression in the PRISM drug screening data (Figure 8B). The drug concentration [IC_50_, EC_50_, ln(IC_50_), or ln(EC_50_) value] of five compounds, PD-198306, ZM-306416, selumetinib, PQ-401, and U-0126, had a significant negative correlation with mRNA expression of *CD27*, *CTLA4*, or *PD1*, respectively, in the PRISM drug screening data (Figure 8C–I).

To investigate if the identified compounds can effectively inhibit the growth of NSCLC cells, the average IC_50_ and EC_50_ values in the CCLE NSCLC cell lines (*n* = 135) of the drugs available in PRISM were examined (Table 3). PD-0325901 (Figure 8J) and dasatinib had small average IC_50_ and EC_50_ values in the PRISM drug screening data, implying their potential to inhibit the growth of NSCLC cells with a safe dose.

### 2.8. Potential Oncogenes and Tumor Suppressor Genes

To identify potential oncogenes and tumor suppressor genes among the seven-gene panel, EMT genes, the intracellular innate immune response (IIIR) genes, and all the intermediate genes in previously identified networks (Figure 5, Figure 6 and Figure 7), genes that were differentially expressed between tumors and NATs and were significantly associated with patient survival in LUAD proteomics data (*n* = 103) were selected [33]. Genes that had significantly higher protein expression (*p* < 0.05; two-sample *t*-tests) in tumors and were survival-hazardous (*p* < 0.05, hazard ration [HR] > 1; univariate Cox proportional-hazards model) were identified as potential oncogenes. Genes that had significantly higher protein expression (*p* < 0.05; two-sample *t*-tests) in NATs and were survival-protective (*p* < 0.05, HR < 1; univariate Cox proportional-hazards model) were identified as potential tumor suppressor genes. The identified potential oncogenes included *EIF4G3*, *GAPVD1*, *IKBKB*, *MCM2*, and *RFC4*. The identified potential tumor suppressor genes included *DAG1*, *SLC39A8*, *TMEM173/STING1*, *RDX*, *TJP1*, *CTNNB1*, and *SMC3*. Somatic copy number alterations and their correlations with mRNA, protein, and phosphoprotein of the selected genes were extracted from Xu et al. [33]. Detailed information is provided in Supplementary File S3.

## 3. Discussion

This study further validated the seven-gene panel in patient recurrence risk stratification using proteomic profiles in early-stage NSCLC patients. In addition, we showed that the seven-gene panel can accurately classify tumors from NATs on both RNA sequencing and proteomic platforms, suggesting its potential diagnostic implications for NSCLC. These findings warrant further clinical studies on liquid biopsies in the early detection of NSCLC. Blood-based assays for predicting NSCLC risk and metastasis are important for thoracic surgeons in clinical decision-making, given that the current accepted benign rate is 5% in surgery and 20–30% in biopsies [40]. Developing minimally invasive biomarker-based assays will reduce unnecessary surgeries and biopsies on patients who do not have lung cancer.

In this study, we further extended our previous findings on the prognostic implication of the seven-gene signature for NSCLC patient survival using proteomics/RNA-Seq data of Xu’s LUAD (*n* = 103) cohort and RNA-Seq data of TCGA-NSCLC patient cohort (*n* = 923). From the available expression data, we found that ABCC4 was positively associated with patient survival in both Xu’s LUAD protein and TCGA-NSCLC RNA-Seq data. DAG1 was positively associated with patient survival at both mRNA and protein levels, while CCL19 and SLC39A8 showed similar associations at the protein level in Xu’s LUAD dataset. At the same time, CD27 mRNA expression was associated with worse patient outcomes. It is noteworthy that the combined protein expression score of ABCC4, DAG1, and SLC39A8 efficiently stratified patient outcome (*p* = 0.0013, HR: 8.378 [1.774, 39.57]; Figure 1A), suggesting that these three proteins could potentially be used as prognostic markers in NSCLC. Furthermore, we showed that ABCC4, DAG1, and SLC39A8 proteins are significantly downregulated in tumors compared to NATs (Figure 2), suggesting that they may play the role of tumor suppressors.

Expression of *ZNF71* was only available at the mRNA level in the TCGA dataset, and we found only a trend for its negative association with patient survival (Figure 3A), consistent with our previous results in RNA-Seq dataset GSE81089 of NSCLC tumors (*n* = 197) [19]. Based on its structure-inferred function, we hypothesized that *ZNF71* could be involved in the suppression of endogenous transposable elements (TEs) expression, which is often activated in cancer and can trigger an innate immune response. We found that overexpression of ZNF71 in A549 lung adenocarcinoma cells resulted in the downregulation of multiple components of the intracellular intrinsic and innate immune systems, including dsRNA (OAS1) and dsDNA (STING, pTBK1) sensors and viral restriction factors (TRIM5, SAMDH1) (Figure 4). Activation of dsRNA and dsDNA sensors often leads to induction of type I interferons, which can later bridge to adaptive immune response. In particular, type I interferons have been shown to influence the maturation and migration of DC cells, which are important for the cross-priming of NK and CD8+ T cells in the tumor microenvironment [34]. Therefore, we calculated the DC xCell score in the TCGA dataset and combined it with *ZNF71* expression levels. Interestingly, a high expression of *ZNF71* and a low DC xCell score were associated with worse patient outcomes (Figure 3B). *ZNF71* gene expression is positively associated with immune infiltration including DC and CD8+ T cells in TCGA NSCLC tumors as we previously reported [15]. These data indicate a potential interplay between *ZNF71* expression in NSCLC and anti-tumor immune response. Thus, future studies should be aimed at the identification of *ZNF71* targets and establishing a mechanistic link between *ZNF71*, innate immune response, and ICI therapy outcomes.

To identify potential new and repositioning medication candidates, we developed mechanisms of action to improve therapy response, extend patient survival, decrease proliferation, and reverse EMT. Therapeutic targets were identified from pan-sensitive genes and pan-resistant genes from the following list: (1) the seven-gene panel, (2) selected epithelial genes and mesenchymal genes, (3) IIIR genes (Figure 4), and (4) all the genes in the *ZNF71*-IIIR gene association networks (Figure 5, Figure 6 and Figure 7). These genes are relevant to *ZNF71.* Direct targets of *ZNF71* will be identified from RNA-sequencing of NSCLC cell lines after *ZNF71* knockdown/overexpression, which is our ongoing research.

Several MEK1/2 inhibitors were selected as potential targeted therapy also with an inhibitory effect on ICIs for treating NSCLC. Selumetinib is an FDA-approved drug to treat neurofibromatosis type 1 with symptomatic, inoperable plexiform neurofibromas. It is also a designated orphan drug for treating thyroid cancer. Selumetinib is being investigated as a secondary therapy for treating late-stage, metastatic, Kras-mutant NSCLC in several trials [41]. Compared with various combination therapies, chemo-, and immune therapy, selumetinib does not have superior efficacy but does have a better safety profile in treating NSCLC [42]. AS-703026 (pimasertib) has a clinical activity of phosphorylated extracellular signal-regulated kinase (pERK) inhibition in peripheral blood mononuclear cells in patients with locally advanced/metastatic melanoma, particularly BRAF- and NRAS-mutated tumors at clinically relevant doses in a phase I study [43]. In a phase I trial of patients with solid tumors, pimasertib inhibited pERK and the recommended phase II dose (RP2D) was defined as 60 mg bid [44]. PD-198306, an orally active inhibitor of MEK1/2, acts as a potent mitochondrial protonophore and uncouples oxidative phosphorylation [45]. PD-198306 is being studied in rabies virus infection [46], neuropsychiatric disorders [47,48], breast cancer [49], and osteoarthritis [50] research. Resulting from direct inhibition of MEK1 and MEK2, U-0126 inhibits endogenous promoters containing activator protein 1 (AP-1) response elements but does not affect genes lacking an AP-1 response element in their promoters [51]. U-0126 inhibits anchorage-independent growth of Ki-ras-transformed rat fibroblasts by concurrently suppressing both ERK and mammalian targets of rapamycin (mTOR)-p70(S6K) pathways, and sensitizes human breast cancer MDA-MB-231 and HBC-4 cells to anoikis [52].

Among other selected compounds with an in vitro inhibitory effect on ICIs, ZM-306416 is a VEGFR antagonist and a potent inhibitor of EGFR function [53]. As an inhibitor of placental growth factor (PGF) receptor FLT1, ZM-306416 impaired trophoblast proliferation and migration in fetal growth restriction [54]. PQ-401 is an IGF-1R inhibitor that induces apoptosis and inhibits in vitro viability, proliferation, and mobility of U87MG glioma cells and in vivo glioma tumor growth in a mouse xenograft model [55]. In a separate study, PQ-401 inhibited osteosarcoma cell proliferation, migration, and colony formation in U2OS and 143B lines [56]. Overall, the above analysis identified several compounds, including PD-198306, U-0126, ZM-306416, and PQ-401, as potential targeted therapy that may also induce immune response for treating NSCLC, which was not known before.

Dasatinib was reported as a potential repositioning drug for treating NSCLC in our previous publication [15]. PD-0325901 was used to treat refractory NSCLC patients but did not meet its primary endpoint in an open-label, phase II study [57]. Combinations of indirubin and arylidene derivatives showed antimetastatic effects on human NSCLC A549 and NCI-H460 cells [58]. Saracatinib, an orally available inhibitor of Src kinases, improved progression-free survival in a subset of patients with advanced, platinum-pretreated NSCLC in phase II clinical trial [59]. BMS-754807 alone reduced cell survival and wound closure and enhanced apoptosis in human NSCLC A549 and NCI-H358 cells, particularly in NSCLC cells expressing high levels of IGF-IR [60]. In addition, BMS-754807 enhanced cisplatin and carboplatin in A549 cells [60]. A combination of trametinib and bosutinib can synergistically suppress the growth of NSCLC by inhibiting both the mitogen-activated protein kinase (MAPK) and proto-oncogene tyrosine-protein kinase (SRC) pathways, suggesting the potential for treating NSCLC, especially in the treatment of erlotinib-resistant NSCLC [61]. Maintenance therapy of adding linsitinib to erlotinib did not improve PFS or OS in non-progressing NSCLC patients in phase II randomized trial [62]. In a separate phase II study, adding linsitinib to erlotinib resulted in worse patient outcomes compared with erlotinib alone, suggesting that biomarkers are needed to select responding patients [63]. Morphine, a µ-opioid receptor (MOR) agonist, promoted the growth of NSCLC H460 cells both in vitro and in vivo; a higher morphine dosage shortens the survival time of patients with lung cancer [64]. Treatment with the Src inhibitor protein phosphatase 1 (PP-1) and the MOR antagonist methylnaltrexone (MNTX) decreased the phosphorylation induced by morphine. Furthermore, the antiapoptotic impact of morphine on NSCLC cells was reversed by MNTX, PP-1, and the PI3K/AKT inhibitor deguelin. Lapatinib (EGFR and HER2 tyrosine kinase inhibitor (TKI)), gefitinib (EGFR TKI), ZD4054 or BQ-123 (ETAR antagonist), GM6001 (matrix metalloprotease inhibitor), PP-2 (Src inhibitor) or Tiron (superoxide scavenger) all inhibited the increase in EGFR and HER2 transactivation induced by the addition of ET-1 to NSCLC cells [65]. These results indicate that our AI pipeline is capable to select relevant compounds for further clinical studies.

Among the identified potential NSCLC oncogenes, a reduction in the germline copy number of *EIF4G3* is linked to breast cancer susceptibility in the Japanese population [66]. IkappaB kinase (IKK) promotes tumorigenesis via inhibiting forkhead *FOXO3a* which can be reversed by *FOXO3a* [67]. Conditional suppression of *IKBKB* inhibits melanoma tumor development in mice, and *IKBKB*-mediated NFkB activity is required in mutant *Hras*-initiated tumorigenesis [68]. *IKBKB* also promotes osteosarcoma cancer progression [69]. Minichromosome maintenance proteins (MCMs) are essential in DNA replication, genomic stability, and cell proliferation [70,71]. MCMs, in particular, *MCM2* and *MCM4*, are potential biomarkers to identify high-risk NSCLC patients [72]. *MCM2* was significantly overexpressed in almost all human cancers/subtypes in TCGA and was associated with tumor mutation burden, tumor stage, immune therapy response, immune infiltration, and poor patient prognosis [73]. *RFC4* is frequently overexpressed in colorectal cancer (CRC), and *RFC4* overexpression is associated with tumor progression and shorter patient survival, possibly due to RFC4-mediated cell cycle arrest and the regulation of CRC cell proliferation [74]. *RFC4*, along with other genes and microRNAs, might promote osteosarcoma initiation and development [75]. *GAPVD1*, a cytoplasmic trafficking factor, is involved in the regulation of the mammalian circadian clock [76]. Mutations in *GAPVD1* and other genes, estrogen- and growth factor-dependent regulation are involved in both transcriptional and post-transcriptional dysregulation of syndecan-4 in breast cancer [77]. Together, our identified NSCLC oncogene genes are supported by the literature.

Among the identified potential tumor suppressor genes, *DAG1* was co-deleted with the Von Hippel Lindau (VHL) tumor suppressor gene in clear cell renal cell carcinoma [78]. In glioblastomas, *DAG1* correlated with tumor grade, and the patient group with higher expression of *DAG1* survived for a shorter time than the patient group with lower expression of *DAG1* [79]. These results suggest that *DAG1* may have different functions in tumor initiation and progression in different cancer types. *SLC39A8* is responsible for pasting zinc to the cytoplasm when zinc is depleted, for maintaining many critical biological processes. *SLC39A8* suppresses the progression of clear cell renal cell carcinoma [80]. *TMEM173/STING1* was expressed higher in normal samples in lung adenocarcinoma, lung squamous carcinoma, prostate adenocarcinoma, uterine corpus endometrial carcinoma, but was expressed higher in tumor tissues in colorectal carcinoma, kidney renal clear cell carcinoma, stomach adenocarcinoma, and thyroid adenocarcinoma [81]. *MTA1*-induced inhibition of TJP1 protein co-localized in the cytoplasm and membrane of NSCLC cells leads to weakened cell junctions and changes in the adhesion, migration, and invasion capabilities of cells, putatively promoting the invasion and metastasis of NSCLC [82]. β-catenin/*CTNNB1* is an intracellular scaffold protein. Aberrant CTNNB1 signaling is one of the fundamental processes in many human cancers [83,84]. Both gain-of-function and loss-of-function *CTNNB1* mutations are found in multiple human cancer types [85]. *SMC3* haploinsufficiency accelerates lymphomagenesis in mice with constitutive *BCL6* expression and is considered a putative tumor suppressor for germinal center B cells [86]. *RDX* knockdown increased the intracellular SN-38 concentration, indicating enhanced anti-tumor activity, in human clear cell renal cell carcinoma Caki-1 cells [87]. To date, the literature supports the tumor suppressor functions of *DAG1*, *SLC39A8*, *TMEM173/STING1*, *TJP1*, *CTNNB1*, and *SMC3*, but not *RDX*.

## 4. Materials and Methods

### 4.1. Non-Small Cell Lung Cancer (NSCLC) Patient Cohorts

This study obtained NSCLC patient sample data from public resources including Xu et al. [33] and The Cancer Genome Atlas (TCGA Research Network: https://www.cancer.gov/tcga, accessed on 28 April 2021). The primary lung adenocarcinoma (LUAD) cohort collected samples from 103 randomly selected treatment-naïve Chinese patients between 2010 and 2016 from Xu et al. [33]. Proteomics data of 103 paired LUAD tumors and non-cancerous adjacent tissues (NATs), and RNA sequencing data of 51 paired LUAD tumors and 49 NATs were used in this study. RNA sequencing data of the TCGA NSCLC patient cohort, i.e., TCGA-LUAD (*n* = 515) and TCGA-LUSC (*n* = 501), with patient clinical information were downloaded from an openly accessible entry LinkedOmics (http://www.linkedomics.org, accessed on 28 April 2021) [88].

### 4.2. xCell

The xCell (https://xcell.ucsf.edu/, accessed on 12 July 2022) tool [89] was used to predict the levels for 64 immune and stroma cell types based on gene expression data. The xCell scores for patient samples were calculated using single-sample gene set enrichment analysis (ssGSEA) to analyze the immune microenvironment. Low xCell scores indicated the cell type had similar levels across all samples, whereas high xCell scores indicated the cell type had different levels across all samples

### 4.3. Weka

The Weka software (Version 3.8.6) [90] was utilized to conduct machine learning classifier approaches to differentiate between tumors and NATs with selected genes in Xu’s LUAD RNA sequencing and proteomics data [33]. Commonly used machine learning classification methods, including decision tree, k-nearest neighbors (KNN), logistic regression, naïve Bayes, random forests, support vector machine (SVM), and radial basis function (RBF) network, were used. Ten-fold cross-validation was applied in each session.

### 4.4. Cell Lines

A549 cells (kind gift of Dr. Ivan Martinez, WVU) were grown in DMEM (Corning, Corning, NY, USA, cat. #15-018-CV) supplemented with 10% FBS (HyClone, Logan, UT, USA), 2 mM L-glutamine (Corning, cat. # 25-005-CI), and 1×Antibiotic Antimycotic Solution (Corning, cat. # 30-004-CI). All cells were maintained at 37C in a 5% CO_2_ incubator.

### 4.5. Vector Construction and Lentiviral Transduction

ZNF71 KRABless isoform was PCR-amplified from cDNA obtained from MDA231 cells using PfuUltraII DNA polymerase using primers #5ZNF71 ex1-Mun: 5′-AGAG**CAATTG**ATGGCTGCTCAGCTGC-3′ and #3ZNF71-X: 5′-AGA**CTCGAG**TCAGGTGTGAATCCGCAG-3′, and cloned into Dox-inducible pLUT lentiviral vector [91] using EcoRI and XhoI cloning sites. To generate the KRAB isoform, a 397 bp long KRAB containing 5′-terminal fragment was synthesized at GenScript and cloned into pLUT-ZNF71-KRABless using NheI and EcoRI sites. The cloned cDNAs were sequenced to verify the absence of mutations.

Lentiviral particles were packaged in HEK293T cells (RRID:CVCL_0063) following calcium phosphate cotransfection of constructed pLUT-ZNF71 vectors, psPAX2 (Addgene, Watertown, MA, USA, 12260), and pCMV-VSV-G (Addgene, 8454) as previously described [91]. pLUT vector expressing turbo red fluorescent protein (RFP) was used as control. After two rounds of infection, transduced A549 cells were selected in puromycin (1 ug/mL) containing media for at least 5 days. Ectopic ZNF71 expression was induced by culturing cells in media containing 0.5 ug/mL doxycycline for 7 days.

### 4.6. Western Blot

Whole-cell lysates were prepared in nonreducing Laemmli buffer as described in [91]. Protein concentration was quantified by Pierce BCA Protein Assay (ThermoFisher, Waltham, MA, USA, cat # 23225). Lysates with an equal amount of total protein were separated on 4 to 12% Bis-Tris NuPAGE Novex gels and transferred to a polyvinylidene difluoride (PVDF) membrane. Protein bands were detected using standard chemiluminescence techniques using GE Healthcare Amersham Imager 680.

The following antibodies were used in Western blotting: ZNF71 (GeneTex, Irvine, CA, USA, cat. # GTX116553), from Cell Signaling Biotechnology (Danvers, MA, USA), STING (cat. #13647), TBK1 pSer172 (cat. #5483), TBK1 (cat. #3504), OAS1 (cat. #14498), RNase L (cat. #27281), TRIM5α (cat. #14326), SAMHD1 pThr592 (cat. #89930), and GAPDH (Millipore, Burlington, MA, USA, MAB374).

### 4.7. Boolean Implication Network

The Boolean implication network algorithm [92,93,94] was used in this study to generate gene associate networks. Details of the algorithm were included in our previous publications [15,95]. Thresholds of scope and precision for filtering the implication rules were computed based on a one-tailed *z*-score of 1.64 (95% level of significance).

Xu’s LUAD cohort [33] was used to construct the gene association networks. Each gene’s expression was divided into three categories: under-expressed (–1), normal (0), and over-expressed (1). The categorization was based on the distribution of the selected housekeeping genes (*B2M*, *ESD*, *FLOT2*, *GAPDH*, *GRB2*, *HPRT1*, *HSP90AB1*, *LDHA*, *NONO*, *POLR2A*, *PPP1CA*, *RHOA*, *SDCBP*, and *TFRC*) [14,96,97,98]. The percentage of under-expressed and over-expressed samples for all the housekeeping genes was fixed to be 30% in each dataset. Standard deviations were calculated for the normal range based on the housekeeping genes and applied to the rest genes. The numbers of standard deviation used for each dataset were: 0.68 for RNA sequencing data of LUAD tumors; 0.75 for RNA sequencing data of NATs; 0.88 for proteomics and RNA sequencing data of LUAD tumors; and 0.95 for proteomics data and RNA sequencing data of NATs. The networks were visualized with Cytoscape (version 3.9.1) [99].

### 4.8. CRISPR-Cas9 Knockout Assays

Genome-scale CRISPR-Cas9 knockdown data from project Achilles [100,101] were obtained from the DepMap (release 21Q4) [102]. CRISPR-Cas9 dependency scores of human NSCLC cell lines (*n* = 94) from the DepMap portal (https://depmap.org/portal/download/all/, accessed on 12 September 2022) were used in this study. The CERES method was used to normalize gene-level dependency scores. The median of the normalized dependency scores of common essential genes was −1.0, and that of non-essential genes was 0 in each cell line. In this study, a dependency score of less than −0.5 indicated that the gene had a significant effect on the cell line in CRISPR-Cas9 knockout.

### 4.9. RNAi Knockdown Assays

Genome-wide RNA interference (RNAi) knockdown screening data [103] in project Achilles was analyzed in this study. RNAi dependency scores were processed with the DEMETER2 v6 algorithm [103] to distinguish between on- and off-target effects. The dependency scores of human NSCLC cell lines (*n* = 92) were obtained from the DepMap portal (https://depmap.org/portal/download/all/, accessed on 12 September 2022). The median of the normalized dependency scores of the positive control gene set was −1, and that of the negative control gene set was 0. The gene that had a dependency score less than −0.5 was considered as having a significant effect on the cell line in RNAi knockdown.

### 4.10. Pathway Enrichment Analysis Using ToppGene

The ToppFun web tool (https://toppgene.cchmc.org/enrichment.jsp, accessed on 28 June 2022) from the ToppGene suite [104] is an online resource for gene list enrichment analysis. We used the ToppGene application with default parameters (FDR correction, *p*-value cutoff = 0.05, gene limits 1 ≤ *n* ≤ 2000) to perform pathway enrichment analysis.

### 4.11. Cancer Cell Line Encyclopedia (CCLE)

RNA sequencing data of 135 human NSCLC cell lines were obtained from the Cancer Cell Line Encyclopedia (CCLE) in DepMap release 22Q2 (https://depmap.org/portal/download/all/, accessed on 12 September 2022). Proteomics data of 64 human NSCLC cell lines were obtained from Nusinow et al. [105].

### 4.12. Drug Sensitivity in CCLE

The drug sensitivity data of human NSCLC cell lines were taken from multiple resources. The secondary PRISM repurposing dataset [35] was obtained from the DepMap release 19Q4. It has 1448 compounds screened in 499 human cell lines, 84 of which were NSCLC cell lines and were used in this study. The Genomics of Drug Sensitivity in Cancer (GDSC) datasets (GDSC1 and GDSC2 [36,37,38]) were downloaded (https://www.cancerrxgene.org/downloads/bulk_download, accessed on 12 September 2022). A total of 64 NSCLC cell lines from GDSC1 and 58 NSCLC cell lines from GDSC2 were included in this study. Details of the categorization of drug sensitivity in each cell line were included in our previous publications [15,95].

### 4.13. Drug Repurposing Using Connectivity Map (CMap)

The connectivity map (CMap) online tool (https://clue.io/, accessed on 12 September 2022) [31,32] was used to explore functional pathways and potential repositioning of drugs with selected gene expression signatures. Raw connectivity scores higher than 0.9 and a *p*-value < 0.05 were considered significant.

### 4.14. Statistical Methods

Statistical analysis was performed using R software (version 4.1.3) with RStudio (version 2022.02.3 Build 492) [106]. Two sample *t*-tests (two-tailed) were performed for the comparisons of two groups of continuous variables. The independence of categorical variables was evaluated with χ^2^ tests. Principle component analysis (PCA) was used to generate the separation of NATs and LUAD tumors with selected genes. Visualization was carried out in R and Cytoscape. The Kaplan–Meier method was performed to conduct survival analysis and create survival curves. The difference between the survival rates of patient groups was evaluated using log-rank tests. Univariate and multivariate Cox regression analyses were performed to evaluate the prognostic capacity of the studied factors. R packages *survival*, *survminer*, and *ggplot2* were used in survival analysis. Pearson’s correlation coefficients were used to determine the association between the two sample groups. All hypothesis tests were two-sided, and test results with a *p*-value < 0.05 were considered statistically significant.

## 5. Conclusions

This study extended a seven-gene panel for NSCLC prognosis using proteomic profiles. The results also showed that the seven-gene panel can accurately classify NSCLC tumors from NATs on both RNA-sequencing and proteomic platforms, suggesting its diagnostic implications for the early detection of lung cancer. Gene expression of *ZNF71*, a marker within the seven-gene panel, when combined with dendritic cell activities, can further stratify NSCLC into different prognostic groups. *ZNF71* expression is associated with the activities of NK cells and NKT cells. Overexpression of ZNF71 results in decreased expression of multiple components of the intracellular intrinsic and innate immune systems, including dsRNA and dsDNA sensors, confirming a hypothesis that *ZNF71* suppresses the transcription of genomic transposable elements. Multi-omics networks of *ZNF71* and the intracellular innate immune response genes were revealed in NSCLC using a computational Boolean implication network algorithm. From these constructed networks, pan-sensitive and pan-resistant genes to 21 NCCN-recommended drugs for treating NSCLC were selected. We designed mechanisms of action to enhance treatment response, prolong patient survival, inhibit proliferation, and reverse EMT to screen candidates for new and repositioning drugs. PD-198306, U-0126, ZM-306416, and PQ-401 were identified as potential targeted therapy that may also induce immune response for treating NSCLC, which was not known before. Our future research will identify direct targets of *ZNF71* for the development of novel therapeutic strategies to improve NSCLC survival outcomes.

## 6. Patents

The seven-gene prognostic panel was included in the US 2021-0254173 A1. The artificial intelligence methodology for drug discovery was filed under PCT/US22/75136.

## Figures and Tables

**Table 1:** Pan-sensitive and pan-resistant genes to 21 drugs using RNA sequencing data in CCLE NSCLC cell lines (*n* = 135).

Drug	Systemic/Targeted Therapy	Pan-Sensitive Genes	Pan-Resistant Genes
afatinib	EGFR Exon 19 Deletion or L858R/EGFR S768I, L861Q, and/or G719X	*CDT1*, *INCENP*	*IL4I1*, *LRP1*, *FAM156B*, *DCUN1D4*, *ESPN*, *PTPRJ*, *NMNAT2*
alectinib	ALK Rearrangement Positive	*CSNK2A3*, *DYNC1H1*, *PSME1*	*UCP3*, *CDC45*, *THSD7A*, *ANKRD52*, *EIF2A*, *FAM156B*, *KRT8*, *CFAP44*
brigatinib	ALK Rearrangement Positive		*MYOF*, *ABHD10*, *TMTC4*
cabozantinib	RET Rearrangement Positive	*CSNK2A3*	*RNASEL*, *TMTC4*, *SMC2*, *ASB7*, *CFAP44*, *IL17RA*, *ZXDA*, *THSD7A*
carboplatin	Systemic		*ASB7*, *RAB1A*
cisplatin	Systemic	*ZNF507*, *CSNK2A3*, *WDR17*	*ARHGAP12*, *CDC45*, *ESPN*, *EIF2A*, *IL17RA*, *MYOF*, *TMTC4*, *NMNAT2*, *PTPRJ*, *KRT8*
crizotinib	ALK Rearrangement Positive/ROS1 Rearrangement Positive/MET Exon 14 Skipping Mutation	*RFC4*, *CSNK2A3*, *MCM2*, *MDN1*, *CDT1*, *INCENP*	*LRP1*, *IL17RA*
dabrafenib	BRAF V600E Mutation Positive	*FBLN7*, *MAP2*, *CTNNB1*, *RNF128*, *CSNK2A3*, *WDR17*, *ZNF507*	*FAM156B*, *SLC39A8*, *SMC2*, *KRT8*, *NMNAT2*
dacomitinib	EGFR Exon 19 Deletion or L858R/EGFR S768I, L861Q, and/or G719X	*CDT1*	*MAP3K7*, *DCUN1D4*, *LRP1*, *THOC5*, *FAM156B*, *ANKRD52*, *THSD7A*
docetaxel	Systemic	*RFC4*, *ABCC5*, *FBLN7*, *MAP2*	*SLC39A8*, *IL17RA*, *KRT8*, *RAB1A*, *THSD7A*, *DCUN1D4*, *MYOF*, *NMNAT2*
erlotinib	EGFR Exon 19 Deletion or L858R/EGFR S768I, L861Q, and/or G719X	*CSNK2A3*, *PSMB6*, *TGFB2*	*FAM156B*, *ASB7*, *TMTC4*, *NMNAT2*
etoposide	Systemic	*PSMB3*, *CDT1*, *DYNC1H1*, *ZNF507*	*ESPN*, *NMNAT2*, *ZNF324B*, *IL17RA*
gefitinib	EGFR Exon 19 Deletion or L858R/EGFR S768I, L861Q, and/or G719X	*BRMS1L*, *CCNA2*, *CDT1*, *INCENP*	*IL4I1*, *ABHD10*, *FAM156B*, *MAP3K7*, *DCUN1D4*, *NMNAT2*, *PTPRJ*, *KRT8*
gemcitabine	Systemic	*MDGA1*, *MDN1*, *ZNF507*, *WDR17*	*KRT8*, *ANKRD52*, *ASB7*, *RAB1A*, *FAM156B*, *IL17RA*, *DCUN1D4*, *IL4I1*, *LRP1*, *NMNAT2*
lorlatinib	ALK Rearrangement Positive/ROS1 Rearrangement Positive	*MDN1*, *PSMB6*	*ANKRD52*, *ESPN*, *RAB1A*
osimertinib	EGFR Exon 19 Deletion or L858R/EGFR S768I, L861Q, and/or G719X	*ABCC5*, *MDGA1*, *TCF20*, *MAP2*, *PSMB6*	*ASB7*, *DCUN1D4*, *THSD7A*
paclitaxel	Systemic	*INCENP*, *CDT1*, *MCM2*, *TCF20*	*RAB1A*, *THOC5*, *ASB7*, *IL17RA*, *NMNAT2*, *KRT8*, *LRP1*, *SLC39A8*, *THSD7A*, *UCP3*
pemetrexed	Systemic	*MCM2*, *CCNA2*	*EIF2A*, *IL17RA*, *MYOF*, *THSD7A*, *ZXDA*, *ARHGAP12*, *LRP1*
trametinib	BRAF V600E Mutation Positive	*CTNNB1*	*RNASEL*, *IL17RA*, *ANKRD52*, *FAM156B*, *AP2S1*, *ARHGAP12*, *SRRM2*, *UCP3*, *ZXDA*, *TMTC4*, *DCUN1D4*, *NCOR1*, *RAB1A*
vemurafenib	BRAF V600E Mutation Positive		*RNASE L*, *IL17RA*
vinorelbine	Systemic	*ABCC5*, *DYNC1H1*, *IRX1*, *TCF20*	*RAB1A*, *SLC39A8*, *KRT8*, *DCUN1D4*, *PTPRJ*, *IL17RA*, *MYOF*, *NMNAT2*

**Table 2:** Pan-sensitive and pan-resistant genes to 21 drugs using proteomics data in CCLE NSCLC cell lines (*n* = 64).

Drug	Systemic/Targeted Therapy	Pan-Sensitive Genes	Pan-Resistant Genes
afatinib	EGFR Exon 19 Deletion or L858R/EGFR S768I, L861Q, and/or G719X	DDX42, RFX7, FAT1, CREBBP	IL4I1, KRT8, CDCA4, DIXDC1
alectinib	ALK Rearrangement Positive	DDX42, ZNF507	CCNA2, KIF14, CDK1, DAG1, ANKRD52, MAP3K7
brigatinib	ALK Rearrangement Positive	FAT1, PTPRJ, PSME1	TEN1, MAP3K7, FBLN7, CDK1
cabozantinib	RET Rearrangement Positive	KANSL1, CHL1, TGFB2	TJP1_G3V1L9, BPTF_E9PE19, NSD1, SYNE2_Q8WXH0_5, RDX, ANKRD52
carboplatin	Systemic		TJP1_G3V1L9
cisplatin	Systemic	MYCL, DDX42, BRMS1L, KANSL1, ZNF507	BPTF_E9PE19, FBLN7, DIP2B, MYOF, CDK1
crizotinib	ALK Rearrangement Positive/ROS1 Rearrangement Positive/MET Exon 14 Skipping Mutation	FAM208A, TOMM7, KANSL1	DAG1
dabrafenib	BRAF V600E Mutation Positive	BRMS1L, RBBP4, ZNF507, DDX42, KANSL1	RDX, ANKRD52, DIP2B, CDK1, DAG1, IL17RA
dacomitinib	EGFR Exon 19 Deletion or L858R/EGFR S768I, L861Q, and/or G719X	PSME1, DDX42, GREB1, RBBP4	MAP2_P11137_4, KRT8, CDC45, CDCA4, IL4I1
docetaxel	Systemic	TOMM7, CREBBP, TCF20, DDX42, FAM208A, THOC5, ZNF507	DYNC1H1, MYOF
erlotinib	EGFR Exon 19 Deletion or L858R/EGFR S768I, L861Q, and/or G719X	RFX7, THOC5, CREBBP	KIF14, CDCA4
etoposide	Systemic	CHL1, FAT1	
gefitinib	EGFR Exon 19 Deletion or L858R/EGFR S768I, L861Q, and/or G719X	DDX42, CREBBP, TCF20, CHL1	CDCA4, IL4I1
gemcitabine	Systemic	DDX42, CREBBP, ZNF507, RBBP4, TOMM7	DIP2B, ANKRD52, CDK1, MAP2_P11137_4, IL17RA, SLC39A8
lorlatinib	ALK Rearrangement Positive/ROS1 Rearrangement Positive	ASB7, PSME1, PIR	CCNA2, MYOF, CDK1, CDC45
osimertinib	EGFR Exon 19 Deletion or L858R/EGFR S768I, L861Q, and/or G719X		CDCA4, EIF4G3_B1AN89, IL4I1, ADARB1, CDC45
paclitaxel	Systemic	CHL1, THOC5, ZNF507, UCHL3	SLC39A8, DIXDC1, DIP2B, MYOF, IL17RA
pemetrexed	Systemic	THOC5	BPTF_E9PE19, RDX, DYNC1H1
trametinib	BRAF V600E Mutation Positive	CHL1, PSME1, TMTC4, JOSD2, PTPRJ, RPS19	RDX, DAG1, IL17RA, MAP3K7
vemurafenib	BRAF V600E Mutation Positive	FAM208A	KRT8
vinorelbine	Systemic	TCF20, UCHL3, RBBP4, THOC5, ZNF507	TJP1_G3V1L9

**Table 3:** Average IC50 and EC50 values of the selected therapeutic compounds in the PRISM dataset. Outliers (drug concentration value > 10) were removed in the calculation of average IC50 and EC50 values.

Src_Set_Id	Compound	Average of IC_50_	Count of IC_50_ (Count of IC_50_ Outliers)	Average of EC_50_	Count of EC_50_ (Count of EC_50_ Outliers)
CP_MEK_INHIBITOR	PD-0325901	0.748	64 (0)	0.386	88 (0)
CP_SRC_INHIBITOR	dasatinib	0.555	92 (0)	0.504	155 (4)
MAP_KINASE_INHIBITOR	PD-98059	4.977	4 (0)	0.607	56 (3)
LEUCINE_RICH_REPEAT_KINASE_INHIBITOR	indirubin	3.548	23 (0)	0.630	160 (7)
CP_MEK_INHIBITOR	selumetinib	2.214	35 (0)	0.647	165 (7)
CP_MEK_INHIBITOR	AS-703026	1.514	100 (2)	0.940	164 (8)
CP_SRC_INHIBITOR	saracatinib	1.733	103 (0)	0.983	170 (6)
CP_IGF_1_INHIBITOR	BMS-754807	1.744	97 (0)	1.022	168 (8)
CP_SRC_INHIBITOR	ZM-306416	2.626	28 (0)	1.081	87 (1)
CP_SRC_INHIBITOR	bosutinib	3.162	46 (0)	1.587	87 (0)
CP_MEK_INHIBITOR	U-0126	4.533	77 (0)	1.868	177 (3)
CP_MEK_INHIBITOR	PD-198306	3.164	67 (1)	2.034	92 (0)
CP_SRC_INHIBITOR	PP-1	3.408	51 (0)	2.079	87 (0)
CP_IGF_1_INHIBITOR	linsitinib	4.251	64 (1)	2.088	146 (9)
CP_MEK_INHIBITOR	PD-184352	3.988	47 (1)	2.190	89 (0)
CP_MEK_INHIBITOR	MEK1-2-inhibitor	4.197	44 (7)	2.200	84 (1)
IGF-1_INHIBITOR	PQ-401	5.344	30 (0)	2.204	79 (1)
CP_SRC_INHIBITOR	PP-2	3.187	64 (0)	2.484	79 (1)

## Data Availability

Links to publicly archived datasets analyzed in this study are provided in the Section 4.

## Supplementary Materials

The following supporting information can be downloaded at: https://www.mdpi.com/article/10.3390/ijms232314978/s1.
