# Supplementary material for: Multi-Omics Immune Interaction Networks in Lung Cancer Tumorigenesis, Proliferation, and Survival
Source: Int J Mol Sci. 2022 Nov 29;23(23):14978. doi: 10.3390/ijms232314978 (PMC9738413; doi:10.3390/ijms232314978)
Supplement: Supplementary file 1 [file ijms-23-14978-s001.zip › Supplementary File S1.pdf]

Table S1. Machine learning classification of tumors vs. NATs in RNA sequencing data of lung adenocarcinoma patients (*n*=51) from Xu et al. Genes used in these classification methods included *ABCC4, CCL19, CD27, DAG1, FUT7*, and *ZNF71*. Classification methods were performed with Weka in 10-fold cross-validation.

	Decision tree	KNN	Logistic regression	Naïve Bayes	Random forests	SVM	RBF Network
Sensitivity	0.765	0.824	0.824	0.745	0.882	0.961	0.824
Specificity	0.714	0.755	0.837	0.939	0.837	0.490	0.898
PPV	0.736	0.778	0.840	0.927	0.849	0.662	0.894
NPV	0.745	0.804	0.820	0.780	0.872	0.923	0.830
Overall Accuracy	0.740	0.790	0.830	0.840	0.860	0.730	0.860
ROC Area	0.759	0.789	0.915	0.920	0.927	0.725	0.896
Odds Ratio	8.13	14.39	23.92	44.82	38.44	23.52	41.07

Table S2. Machine learning classification of tumors vs. NATs in proteomics data of lung adenocarcinoma patients (*n*=103) from Xu et al. Genes used in these classification methods included ABCC4, DAG1, and SLC39A8. Classification methods were performed with Weka in 10-fold cross-validation.

	Decision tree	KNN	Logistic regression	Naïve Bayes	Random forest	SVM	RBF Network
Sensitivity	0.800	0.822	0.867	0.911	0.822	0.867	0.889
Specificity	0.967	0.944	0.956	0.922	0.922	0.978	0.956
PPV	0.923	0.881	0.907	0.854	0.841	0.951	0.909
NPV	0.906	0.914	0.935	0.954	0.912	0.936	0.945
Overall Accuracy	0.911	0.904	0.926	0.919	0.889	0.941	0.933
ROC Area	0.897	0.879	0.957	0.967	0.959	0.922	0.949
Odds Ratio	116	78.63	139.75	121.54	54.84	286	172

Table S3. Proliferation and differential expression status of the seven-gene panel and intracellular immune response (IIIR) genes. NA – Not available in the data.

Type	Gene	Percentage of significant (<– 0.5) dependency score in RNAi screening	Percentage of significant (<– 0.5) dependency score in CRISPR-Cas9 screening	Xu's LUAD protein $\log_{10}$ transformed	Xu's LUAD RNA sequencing
7-gene	ABCC4	0.00%	0.00%	Higher in normal	Higher in tumor
7-gene	CCL19	0.00%	0.00%	Higher in tumor	Higher in tumor
7-gene	CD27	0.00%	0.00%	Not significant	Higher in tumor
7-gene	DAG1	0.00%	0.00%	Higher in normal	Not significant
7-gene	FUT7	0.00%	0.00%	NA	Higher in normal
7-gene	SLC39A8	0.00%	0.00%	Higher in normal	NA
7-gene	ZNF71	0.00%	0.00%	NA	Not significant
IIR gene	AIM2	3.26%	0.00%	Not significant	NA
IIR gene	CDK1	98.91%	100.00%	Not significant	Higher in tumor
IIR gene	EIF2A	0.00%	0.00%	Higher in tumor	Higher in tumor
IIR gene	EIF2AK2	0.00%	0.00%	Higher in tumor	NA
IIR gene	IFNA16	0.00%	0.00%	NA	NA
IIR gene	IFNA17	0.00%	NA	NA	NA
IIR gene	IFNA21	0.00%	NA	NA	NA
IIR gene	IFNA22P	0.00%	0.00%	NA	NA
IIR gene	IFNA4	0.00%	0.00%	NA	NA
IIR gene	IFNA5	15.22%	0.00%	NA	NA
IIR gene	IFNAR1	0.00%	0.00%	Not significant	Higher in normal
IIR gene	IFNAR2	0.00%	0.00%	Not significant	Higher in tumor
IIR gene	IFNE	5.43%	0.00%	NA	Not significant
IIR gene	IFNG	0.00%	0.00%	NA	NA
IIR gene	IFNG-AS1	0.00%	0.00%	NA	Higher in tumor
IIR gene	IFNGR1	0.00%	0.00%	Higher in normal	Higher in normal
IIR gene	IFNGR2	0.00%	0.00%	NA	Higher in tumor
IIR gene	IFNK	0.00%	0.00%	NA	NA
IIR gene	IFNL1	0.00%	0.00%	NA	NA
IIR gene	IFNL3	0.00%	0.00%	NA	NA
IIR gene	IFNLR1	0.00%	0.00%	NA	Not significant
IIR gene	IFNW1	1.09%	0.00%	NA	NA
IIR gene	IKBKB	0.00%	0.00%	Higher in tumor	Higher in tumor
IIR gene	IRF3	1.09%	0.00%	Higher in tumor	Higher in normal
IIR gene	IRF7	2.17%	0.00%	Not significant	Higher in tumor
IIR gene	JUN	21.74%	12.77%	Not significant	Higher in normal
IIR gene	MAP3K7	0.00%	5.32%	Higher in tumor	Higher in tumor
IIR gene	MB21D1	1.37%	NA	Higher in tumor	NA
IIR gene	OAS1	0.00%	0.00%	Not significant	Higher in tumor
IIR gene	PFKL	0.00%	0.00%	Higher in tumor	NA

IIR gene	RNASEL	1.09%	0.00%	Higher in tumor	Higher in tumor
IIR gene	SAMHD1	1.37%	3.19%	Higher in tumor	Higher in normal
IIR gene	TBK1	1.09%	0.00%	Higher in tumor	Higher in tumor
IIR gene	TMEM173	8.22%	NA	Higher in normal	NA
IIR gene	TRIM28	13.04%	1.06%	Higher in tumor	NA
IIR gene	TRIM5	0.00%	0.00%	Not significant	NA

Table S4. Significantly ($p < 0.05$, connectivity score > 0.9) enriched functional pathways in analysis with CMap.

src_set_id	cell_iname	pert_type	genes
BIOCARTA_AKAPCENTROSOME_PATHWAY	HCC515	TRT_SH.CGS	*AKAP9, CDK1, MAP2, NUP85, PCNT, PKN1, PPP2CA, PRKACB, PRKACG, PRKAG1, PRKAR2A, PRKAR2B, PRKCE, RHOA*
BIOCARTA_BARR_MAPK_PATHWAY	A549	TRT_SH.CGS	*ADCY1, ARRB1, DNM1, GNAS, GRK2, KCNA1, KCNA2, KCNA3, MAP2K1, MAP2K2, MAPK1, MAPK3, PLCB1, RAF1*
BIOCARTA_RB_PATHWAY	A549	TRT_SH.CGS	*ATM, CDC25A, CDC25B, CDC25C, CDK1, CDK2, CDK4, CHEK1, MYT1, RB1, TP53, WEE1, YWHAH*
KD_CYCLIN_DEPENDENT_KINASES	A549	TRT_SH.CGS	*CDK2, CDK4, CDK6, CDK9, CDKL4, CDK10*
KD_CYCLINS	A549	TRT_SH.CGS	*CCNL1, CCND1, CCNA1, CCNH*
KD_INTEGRIN_SUBUNITS_BETA	A549	TRT_XPR	*ITGB1, ITGB4, ITGB5*
KD_PHOSPHOLIPASES	A549	TRT_SH.CGS	*PLCB1, PLA2G2A, PLCG1, PLD2*
OE_NADH_UBIQUINONE_OXIDOREDUCTASE_CORE_SUBUNITS	A549	TRT_SH.CGS	*NDUFS3, NDUFS7, NDUFV2*
OE_PHOSPHOLIPASES	A549	TRT_SH.CGS	*PLCG2, PLA2G12B, PLCB1, PLD1*
PID_VEGF_VEGFR_PATHWAY	HCC515	TRT_SH.CGS	*FLT1, FLT4, KDR, NRP1, NRP2, PGF, VEGFA, VEGFB, VEGFC, VEGFD*
REACTOME_GAP_JUNCTION_DEGRADATION	A549	TRT_SH.CGS	*ACTB, ACTG1, AP2M1, CLTA, CLTB, CLTC, CLTCL1, DAB2, DNM1, DNM2, GJA1, MYO6*
REACTOME_HYALURONAN_UPTAKE_AND_DEGRADATION	A549	TRT_SH.CGS	*CD44, CHP1, GUSB, HEXA, HEXB, HMMR, HYAL1, HYAL2, HYAL3, LYVE1, SLC9A1, STAB2*
REACTOME_TRAF3_DEPENDENT_IRF_ACTIVATION_PATHWAY	A549	TRT_SH.CGS	*CREBBP, DDX58, EP300, IFIH1, IFNB1, IKBKE, IRF3, IRF7, MAVS, RNF135, SIKE1, TBK1, TRAF3, TRIM25, TRIM4*

Table S5. Significantly ($p < 0.05$, connectivity score > 0.9) enriched compound sets in the analysis with CMap.

src_set_id	cell_iname	pert_type	compounds
CP_IGF_1_INHIBITOR	A549	TRT_CP	BMS-536924, BMS-754807, linsitinib
CP_MEK_INHIBITOR	HCC515	TRT_CP	MEK1-2-inhibitor, PD-198306, PD-98059, U-0126, selumetinib, PD-184352, PD-0325901, AS-703026

CP_SRC_INHIBITOR	HCC515	TRT_CP	PP-1, PP-2, ZM-306416, dasatinib, saracatinib, WH-4023, bosutinib
IGF-1_INHIBITOR	A549	TRT_CP	I-OMe-AG-538, tyrphostin-AG-538, PQ-401, BMS-536924, BMS-754807, linsitinib, GSK-1904529A, EI-247
LEUCINE_RICH_REPEAT_KINASE_INHIBITOR	A549	TRT_CP	GW-5074, indirubin, XMD-1150, XMD-885
MAP_KINASE_INHIBITOR	HCC515	TRT_CP	CGP-57380, PD-198306, PD-98059, rottlerin, PD-0325901, XMD-892, XMD-885
PPAR_RECEPTOR_ANTAGONIST	HCC515	TRT_CP	bisphenol-a, mifobate, GW-6471, GW-9662, T-0070907, GSK-0660
SULFONYLUREA	HCC515	TRT_CP	glipizide, glibenclamide, gliquidone
VASOPRESSIN_RECEPTOR_ANTAGONIST	A549	TRT_CP	relcovaptan
